# PCSK9 is not secreted from mature differentiated intestinal cells

**DOI:** 10.1016/j.jlr.2021.100096

**Published:** 2021-07-17

**Authors:** François Moreau, Aurélie Thédrez, Damien Garçon, Audrey Ayer, Thibaud Sotin, Wieneke Dijk, Claire Blanchard, Gilliane Chadeuf, Lucie Arnaud, Mikael Croyal, Laurianne Van Landeghem, Melissa Touvron, Xavier Prieur, Anna Roubtsova, Nabil Seidah, Annik Prat, Bertrand Cariou, Cedric Le May

**Affiliations:** 1 L'institut du thorax, INSERM, CNRS, UNIV NANTES, Nantes, France; 2 Laboratory of Biochemical Neuroendocrinology, Institut de Recherches Cliniques de Montréal, Affiliated to the Université de Montréal, Montreal, Canada; 3 L'institut du thorax, INSERM, CNRS, UNIV NANTES, CHU Nantes, Nantes, France; 4 CRNH-O Mass Spectrometry Core Facility, Nantes, France; 5 Department of Molecular Biomedical Sciences, College of Veterinary Medicine, North Carolina State University, Raleigh, NC, USA

**Keywords:** PCSK9, intestine, cholesterol metabolism, lipoprotein metabolism, Caco-2 cell, liver-specific knockout, portal blood, systemic blood, Ussing chambers, 3′DGE, 3′ digital gene expression, DEGs, differentially expressed genes, endoH, endoglycosidase H, PCSK9 LivKO, mouse model lacking PCSK9 specifically in hepatocytes, PCSK9, proprotein convertase subtilisin/kexin type 9, PNGase F, N-glycosidase F, PPL, postprandial lipemia, TICE, trans-intestinal cholesterol excretion, WB, Western blot

## Abstract

Proprotein convertase subtilisin/kexin type 9 (PCSK9) promotes lysosomal degradation of the LDL receptor and is a key regulator of cholesterol metabolism. After the liver, the small intestine is the second organ that highly expresses PCSK9. However, the small intestine's ability to secrete PCSK9 remains a matter of debate. While liver-specific PCSK9-deficient mice present no PCSK9 in systemic blood, human intestinal Caco-2 cells can actively secrete PCSK9. This raises the possibility for active intestinal secretion via the portal blood. Here, we aimed to determine whether enterocytes can secrete PCSK9 using in vitro, ex vivo, and in vivo approaches. We first observed that PCSK9 secretion from Caco-2 cells was biphasic and dependent on Caco-2 maturation status. Transcriptional analysis suggested that this transient reduction in PCSK9 secretion might be due to loss of SREBP2-mediated transcription of *PCSK9*. Consistently, PCSK9 secretion was not detected ex vivo in human or mouse intestinal biopsies mounted in Ussing chambers. Finally, direct comparison of systemic versus portal blood PCSK9 concentrations in WT or liver-specific PCSK9-deficient mice confirmed the inability of the small intestine to secrete PCSK9 into the portal compartment. Altogether, our data demonstrate that mature enterocytes do not secrete PCSK9 and reinforce the central role of the liver in the regulation of the concentration of circulating PCSK9 and consequently of cellular LDL receptors.

Proprotein convertase subtilisin/kexin type 9 (PCSK9), a secretory protein discovered in 2003 (1, 2), escorts the LDL receptor (LDLR) toward lysosomal degradation. Genetic and interventional clinical studies with PCSK9 monoclonal antibodies have demonstrated that PCSK9 inhibition efficiently reduces plasma LDL cholesterol and represent an efficient and safe strategy to protect against cardiovascular diseases (reviewed in (3)). Although therapeutic PCSK9 inhibition has not been associated to date with clinically meaningful adverse effects, there is still much to be learned about PCSK9 biology, notably its role beyond the liver. Indeed, only few studies have explored extrahepatic functions of PCSK9 (reviewed in (4, 5)).

The development of a mouse model lacking PCSK9 specifically in hepatocytes (PCSK9 LivKO) indicated that the liver is responsible for two-thirds of the hypocholesterolemia observed in whole-body PCSK9-deficient mice (KO), suggesting that PCSK9 also controls cholesterol homeostasis through extrahepatic pathway(s) (6). Beyond the liver, the small intestine highly expresses PCSK9 (1) and plays a crucial role in cholesterol homeostasis by regulating dietary lipid absorption, chylomicrons secretion, bile acid reabsorption, HDL production, as well as the trans-intestinal cholesterol excretion (TICE) pathway (reviewed in (7)). Our group previously reported that PCSK9 deficiency affects postprandial lipemia (PPL), intestinal apolipoprotein B secretion (8), and TICE (9). However, until very recently (10), it was unclear whether all these effects were due to the absence of plasma PCSK9 or directly to intestinal PCSK9 deficiency.

Whether the intestine is able to secrete PCSK9 at a significant and physiological level remains a matter of debate and has not been clearly demonstrated. In 2008, Zaid *et al.* showed that PCSK9 LivKO mice present an absence of detectable PCSK9 in peripheral blood, thereby suggesting that circulating PCSK9 is exclusively produced by hepatocytes (6). This concept was challenged by a study performed in human Caco-2 cells, showing that these intestinal cells both express and secrete PCSK9 from their basolateral side (11). As PCSK9 concentrations in the portal blood were not measured in the seminal analysis of PCSK9 LivKO mice, it is difficult to completely rule out the possibility that the small intestine can target the hepatic LDLR via the portal vein.

We thus assessed the capacity of intestinal cells to secrete PCSK9 using in vitro, ex vivo, and in vivo approaches.

## Materials and methods

### Human colon biopsies

Nontumoral colon tissue fragments from five patients with colorectal adenocarcinoma were processed according to the French Guidelines for Research on Human Tissues (Agence Nationale d'Accréditation et d'Evaluation en Santé 2001) and were registered with the Direction Générale de la Santé, Ministère de la Santé et des Solidarités (Directorate General of Health–Ministry of Health and Solidarity—DC-2011-1399) after approval by the ethics committees CPP Ouest II of Angers. The study was conducted according to principles of the Declaration of Helsinki and in compliance with International Conference on Harmonisation (ICH)/Good Clinical Practice (GCP) regulations. Informed patient consent was obtained according to the French bioethics law No. 2004-800.

### Animals

C57BL6/J mice, *Ldlr*-deficient mice, and PCSK9 LivKO mice (*Pcsk9f/f*; Tg(Alb-cre)+/0) and their control floxed mice (*Pcsk9f/f*) (6) were, respectively, from Charles River Laboratories (France), from Jackson Laboratories (Maine), and from Nabil Seidah's laboratory (IRCM, Montreal, Canada). Mice had free access to food and water under a 12-h light/12-h dark cycle in a temperature-controlled environment. All animal studies were conducted with age-matched male and female mice (2 months-old) and were approved by the Pays de la Loire ethics committee and the French national veterinary agency under the number 01953.01.

### Caco2 cells culture

Human Caco2 cells were purchased from the ATCC (Molsheim, France). Caco2 cells were maintained in TPP® tissue culture flasks in DMEM containing 4.5 g/L glucose and pyruvate (Gibco™) and supplemented with 10% decomplemented FBS (Corning™), 2 mM glutamine, 100 U/ml penicillin, and 100 mg/ml streptomycin. Once 80% confluence was reached, cells were detached with trypsin-EDTA (Sigma-Aldrich, Saint Quentin Fallavier, France) and diluted for maintenance or for the differentiation protocol. To induce differentiation, Caco2 cells were seeded at a density of 0.1 × 10^6^ cells/0.33 cm^2^ onto polycarbonate micropore membranes (0.4 mm pore size) in Transwell® inserts (Costar, Cambridge, MA). Media from apical and basolateral compartment were changed every two days. The confluency and the setup of the cell monolayer integrity were evaluated by measuring transepithelial electrical resistance with a voltmeter equipped with a chopstick electrode (Millicell ERS; Millipore, Saint-Quentin-en-Yvelines, France). In a first set of experiments, basolateral supernatants were collected every 48 h from day 4 to day 22 of differentiation in Transwell® cell culture inserts. In a second set of experiments, secretion assays were performed during 2 h at days 8 and 16 after seeding: both basolateral/apical supernatants and cells were harvested. Cells were collected from Transwells using trypsin-EDTA 1X (Sigma-Aldrich) and then frozen on dry ice and stored at −80°C. PCSK9 concentrations in cell culture supernatants were measured using the human PCSK9 DuoSet ELISA kit DY3888 from R&D systems (Bio-Techne, Lille, France). Acetone-precipitated apoB and PCSK9 proteins from media were quantified by Western blot (WB).

#### PCSK9 secretion assay from explants in Ussing chambers

Explants of the duodenum, jejunum, ileum, and colon from C57BL6/J and *Ldlr*-deficient mice or of the human colon were placed on sliders and inserted in Ussing chambers (Physiologic Instruments, San Diego, CA). Tissues were incubated at 37°C in an oxygenated Krebs solution (115 mM NaCl, 25 mM NaHCO_3_, 2.4 mM K_2_HPO_4_, 1.2 mM MgCl_2_, 1.2 mM CaCl_2_, and 0.4 mM KH_2_PO_4_) supplemented with 10 mM glucose in the basolateral media or 10 mM mannitol in the apical media. After 2 h of incubation, basolateral and apical media and tissues were collected. PCSK9 and ApoB contents in these samples were analyzed using ELISA kits (CircuLex CY-8078/CY8079, CliniSciences, France; Mabtech 3715-1H-6, France) according to manufacturer's instructions.

#### PCSK9 measurement in various mouse biological compartments

After 14 h of fasting, mice received an olive oil gavage. All mice were anesthetized with a mix of ketamine/xylazine (90/10 mg/kg BW ip) 1 h after gavage. Systemic blood was harvested from the mice tail or cheek. A laparotomy was performed, and bile was diverted and collected by gravimetry. The small intestine was perfused as described in (9). Portal blood, urine, and lymph were harvested from the portal vein, bladder, and mesenteric lymph, respectively. Samples were frozen at −80°C. PCSK9 measurements in plasma from blood samples and in others fluids were performed with ELISA kit CY-8078 from CircuLex.

### WB

Caco2 cells were lysed at 4°C in the buffer containing 50 mM Tris HCl at pH 8, 150 mM NaCl, 1% NP40, 1 mM EDTA, 1% of protease inhibitors (Roche Diagnostics, Mannheim, Germany), and 1% of phosphatase inhibitors (Sigma-Aldrich). Intestinal mucosa was homogenized at 4°C in T-PER™ buffer (Thermo Fisher scientific, Illkirch-Graffenstaden, France) supplemented with 1 mM EDTA and 1% of protease inhibitors. Lysates were centrifuged at 14,000 *g* for 5 min at 4°C, and protein supernatants were recovered. Protein concentrations in samples were determined using Pierce™ BCA protein assay kit (Thermo Fisher Scientific). Proteins from cell lysates, intestinal mucosa lysates, or culture supernatants (after protein precipitation with acetone) were resolved on NuPAGE 4%–12% Bis-Tris gels in NuPAGE MOPS SDS running buffer (Invitrogen®, Thermo Fisher scientific) under reducing conditions for WB analysis. Proteins were transferred onto a 0.2 μm nitrocellulose membrane using the Trans-Blot Turbo transfer system (Bio-Rad, Marnes-La-Coquette, France) and probed with antibodies. We used monoclonal or polyclonal antibodies raised against either human PCSK9 (ab181142 from Abcam, Paris, France/AF3888 from R&D Systems), mouse/rat PCSK9 (AF3995 from R&D Systems), human apoB (AB742 from Merck Millipore), human or mouse beta actin (A5616 or A5451, respectively, from Sigma-Aldrich), and suitable HRP-secondary antibodies purchased from Cell Signaling Technology or R&D systems. Immunoreactive bands were finally revealed using clarity or clarity max ECL Western blotting substrates from Bio-Rad.

#### N-glycosidase digestion

Proteins from Caco2 cell lysates were digested with EndoH (kit P0702L, New England Biolabs (NEB), Evry, France) or N-glycosidase F (PNGase F) (kit P0708L, NEB) according to manufacturer's instructions. Briefly, 20 μg of lysate proteins were heated in a glycoprotein-denaturing buffer at 100°C for 10 min. Samples were then incubated for 1 h at 37°C under agitation in the presence or absence of 1000 U of PNGase F or 2500 U of endoglycosidase H (endoH) in GlycoBuffer 2 plus NP40 or GlycoBuffer 3, respectively. Samples were next resolved on NuPAGE 8% Bis-Tris gels in MES SDS buffer (Invitrogen®) under reducing conditions for PCSK9 analysis by WB.

#### Total RNA extraction, reverse transcription, and real-time quantitative PCR

Total RNA was extracted with the NucleoSpin RNA II kit from Macherey-Nagel (Hoerdt, France). For real-time quantitative PCR, total RNA was reverse-transcribed using M-MLV inverse transcriptase (Invitrogen®). Real-time PCR assays were performed with the MESA Green qPCR MasterMix plus (Eurogentec, Angers, France) using primers described in supplemntal Table S1 with the Applied Biosystems 7900HT System (40 cycles: 5 s at 95°C, 60 s at 60°C, and 5 s at 72°C). Gene expression was quantified by the comparative Ct method, in which the amount of target is expressed as 2-ΔΔCt using human cyclophilin as the house-keeping gene.

#### 3′ Digital gene expression RNA sequencing and comparative analysis

Total RNA from four samples of differentiated Caco2 cells at day 8 and day 16 of culture were extracted as described above. 3′ Digital gene expression (3′DGE) RNA sequencing was next performed by Genomics and Bioinformatics Core Facility GenoBIRD (Nantes, France) as described in (12). For differential 3′ DGE RNA expression analysis, genes with no read in at least four samples were filtered out from analysis. Then, differential gene expression analysis between samples at day 16 and day 8 was performed using DSeq function from DESeq2 library on R Studio software v1.2.5042. Genes with a *P* value adjusted with the Benjamini-Hochberg procedure <0.05 and a fold change ≤ 0.6 or ≥ 0.6 were qualified as differentially expressed genes (DEGs). Normalized counts from DESeq2 analysis were transformed with rLog (regularized log transformation) function from DESeq2 library. rLog-transformed counts were used for variance analysis such as principal components analysis or hierarchical clustering (data not shown). MA plots were constructed from DESeq2 analysis: the mean of normalized counts across all samples for each gene (base mean) was plotted versus the base 2 of logarithm of (mean normalized count at day 16 minus mean normalized count at day 8) (Log2 (fold change)). A clustered heat map was finally constructed with transformed counts of DEGs with pheatmap function from pheatmap library. Cluster trees were computed with the Euclidean distance correlation method. Functional clustering of GO biological process terms enriched for genes either upregulated or downregulated at day 16 was performed with online Metascape software (13). Online STRING software V11.0 was then used to analyze protein-protein interactions for DEGs into functional clusters. An MCL clustering with an inflation parameter of 3 was performed.

#### Statistics

All statistical comparative analyses were performed with the software Prism (GraphPad) using a nonparametric Mann-Whitney test, except for differential 3′DGE RNA expression analysis which was done using R (as described below). A *P* value < 0.05 from the Mann-Whitney test was considered as a significant difference.

## Results

### PCSK9 secretion by Caco2 cells is biphasic and depends on the degree of cellular differentiation into enterocytes

**Fig. 1:**
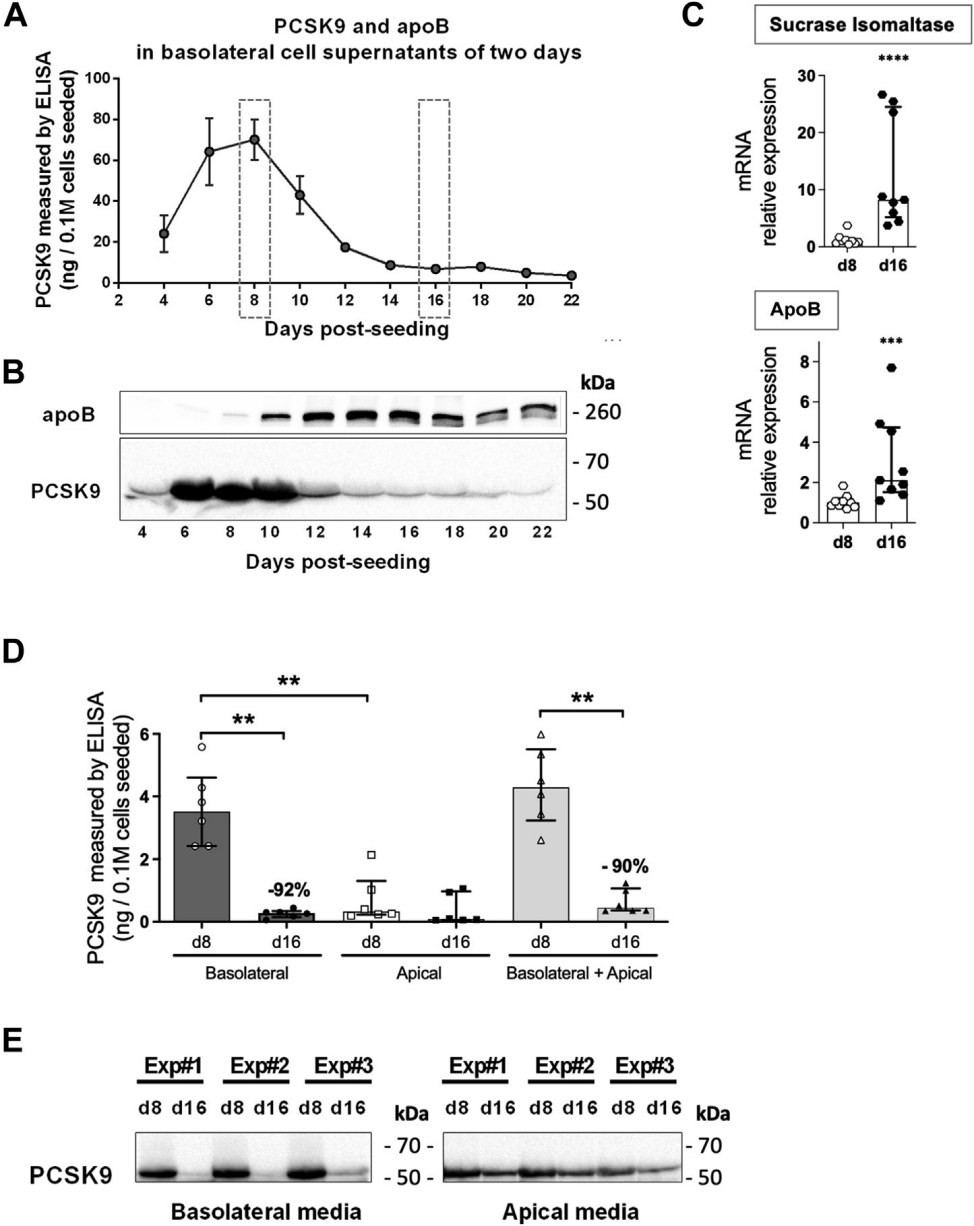
Caco2 cells display a biphasic secretion of PCSK9 at the basolateral side during their differentiation into enterocytes. Caco2 cells were maintained on polycarbonate micropore membranes inserted into Transwells. The medium was changed every two days. A and B: Basolateral supernatants were collected every two days from day 4 to day 22 in two or more independent experiments done in triplicates. A: PCSK9 was measured in supernatants by ELISA. B: Proteins from supernatants (300 μl) were precipitated with acetone, and apoB and PCSK9 were analyzed by Western Blot (WB). C: Caco2 cells were recovered at day 8 (d8) and at day 16 (d16) after a 2-h secretion assay, and mRNAs were extracted. Sucrase isomaltase and ApoB expression relative to that of cyclophilin was analyzed by RT-qPCR in samples from nine independent experiments. Scatter dot plots + bars (median ± interquartile) are presented. ∗∗∗*P*< 0.001; ∗∗∗∗*P*< 0.0001. D and E: Day 8 (d8) and day 16 (d16) after Caco2 cells seeding, the PCSK9 content in supernatants from basolateral and apical sides after a secretion assay of 2 h was measured (C) by ELISA, scatter dot plots + histograms represent median ± interquartile. ∗∗*P* value < 0.01, or analyzed (D) by WB after acetone protein precipitation, the right blot showing apical PCSK9 secretion has been voluntarily overexposed to facilitate band visualization. Results are from six and three independent experiments, respectively. PCSK9, proprotein convertase subtilisin/kexin type 9.

We first investigated the ability of Caco2 cells to secrete PCSK9 in the basolateral compartment every two days during 22 days of culture. As previously reported by Levy *et al.* (11), we confirmed that Caco2 cells are able to secrete PCSK9 (Fig. 1). As shown in Fig. 1A, B, PCSK9 secretion is biphasic and dependent on the extent of Caco2 cell differentiation into enterocytes. Indeed, PCSK9 basolateral secretion increased during the early stages of differentiation to reach a maximum at day 8 and then dramatically decreased during advanced stages of differentiation. As a marker of enterocyte maturation, apoB protein secretion was detectable at day 8, peaked at days 12–16, and remained robust along up to day 22. To further decipher the mechanism responsible for this biphasic PCSK9 secretion, we focused on two specific time points, day 8 and day 16, that we defined as immature and mature enterocyte-like cell stages based on the mRNA expression of two markers of enterocyte maturation, that is, the sucrose isomaltase and the ApoB (Fig. 1C). Global PCSK9 secretion in basolateral and apical media was reduced by 90% in cells at day 16 (median of 0.44 ng/well) compared with that at day 8 (median of 4.29 ng/well). Surprisingly, Caco2 cells were also able to secrete modest amounts of PCSK9 at the apical side of enterocytes. At day 8, this apical secretion was very low compared with the basolateral secretion of PCSK9. By contrast and due to the dramatical decrease of basolateral secretion, apical and basolateral PCSK9 secretion became comparable at day 16 (Fig. 1D). Importantly, whatever the stage of differentiation, only the mature form of PCSK9 (~62 kDa) was detected in either basolateral or apical supernatants at both stages (Fig. 1E), excluding the contribution of endoplasmic reticulum (ER) pro-PCSK9 (~75 kDa) released by death cells. These observations strongly suggest a significantly reduced ability of mature enterocytes to secrete PCSK9.

Caco-2 cells were derived from human colon adenocarcinoma cells. Although they acquired enterocyte-like characteristics when cultured on transwell micromembrane, they certainly differ from mature enterocytes by many aspects. To overpass the limitation of this model and further confirm that PCSK9 secretion is dependent on enterocyte degree of maturation, we measured PCSK9 secretion from a culture of isolated murine primary intestinal epithelial cells (14). Interestingly, PCSK9 secretion was reduced by 40% in epithelial differentiated intestinal cells (i.e., enterocytes, Paneth and goblet cells) compared with intestinal stem cells (supplemental Fig. S1), further validating the hypothesis that enterocyte maturation alters the cellular capacity to secrete PCSK9.

### Intracellular content of PCSK9 decreases with maturation of Caco2 cells

**Fig. 2:**
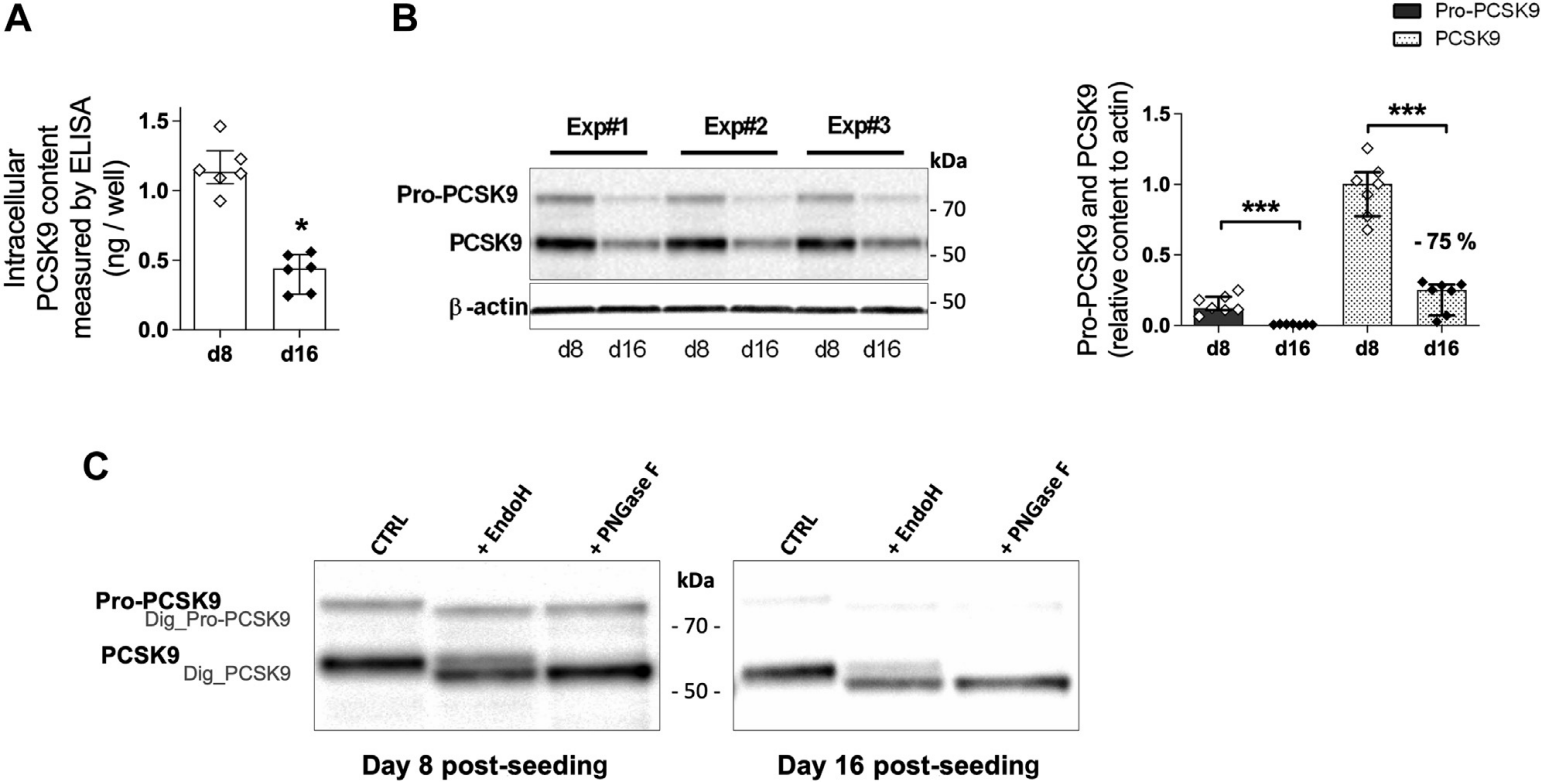
Intracellular PCSK9 content in Caco-2 cells decreases with cell maturation. Differentiated Caco2 cells at d8 and at d16 were recovered after a 2-h secretion assay, and intracellular proteins were extracted from cells. A: Intracellular PCSK9 content (ng extracted from cells) in samples from six independent experiments were measured by ELISA. B: Intracellular pro-PCSK9, PCSK9 and β-actin contents were analyzed by WB. Representative images from three independent experiments are shown (left panel). Pro-PCSK9 and PCSK9 contents relative to b-actin content from seven independent experiments are shown (right panel). C: Cells lysates were denatured and incubated 3 h at 37°C in presence or not of endoH or PNGase F enzymes, followed by WB for PCSK9. Bands corresponding to undigested Pro-PCSK9 (Pro-PCSK9), undigested PCSK9 (PCSK9), digested PCSK9 (Dig-PCSK9), and digested Pro-PCSK9 (Dig-Pro-PCSK9) by enzymes are indicated. One representative result from two independent experiments is shown. Histograms represents the median ± interquartile. ∗*P* value< 0.05, ∗∗∗*P* value <0.001. PCSK9, proprotein convertase subtilisin/kexin type 9; WB, Western blot.

To better understand the mechanism involved in this dramatic decrease of PCSK9 secretion with the maturation of Caco2 cells, we measured intracellular PCSK9 protein content in Caco2 cells by ELISA after 8 and 16 days of culture. PCSK9 content was significantly reduced (−70%) at day 16 (median of 0.44 ng/well) compared with day 8 (median of 1.13 ng/well) (Fig. 2A), whereas no significant difference in the total protein content per well was observed (162 and 173 μg of proteins /well at day 8 and 16, respectively, *P* > 0.05). As estimated by WB analysis (Fig. 2B), both pro-PCSK9 and mature PCSK9 were strongly reduced at day 16 as compared with day 8 (−92% and −75%, respectively). The asparagine 533 of PCSK9 is N-glycosylated in the ER and then modified by complex-type glycans in the Golgi (1). To better understand PCSK9 trafficking during enterocyte differentiation, we compared the intracellular proportion at day 8 and day 16 of mature PCSK9 localized in the ER, sensitive to endoH, and in the Golgi, resistant to endoH. No significant differences were detected between day 8 and 16: 62% of PCSK9 content was in an ER form (endoH sensitive) and 38% was in a mature Golgi form (resistant to endoH) at (Fig. 2C). Similarly, the digestion with the PNGase F that digests both immature and mature glycans resulted in a complete digestion of mature PCSK9 at both stages (Fig. 2C). These data support a reduced PCSK9 content in mature enterocytes compared with immature enterocytes with comparable ER exit and N-glycosylation of PCSK9.

### PCSK9 mRNA levels decrease in mature Caco2 cells

**Fig. 3:**
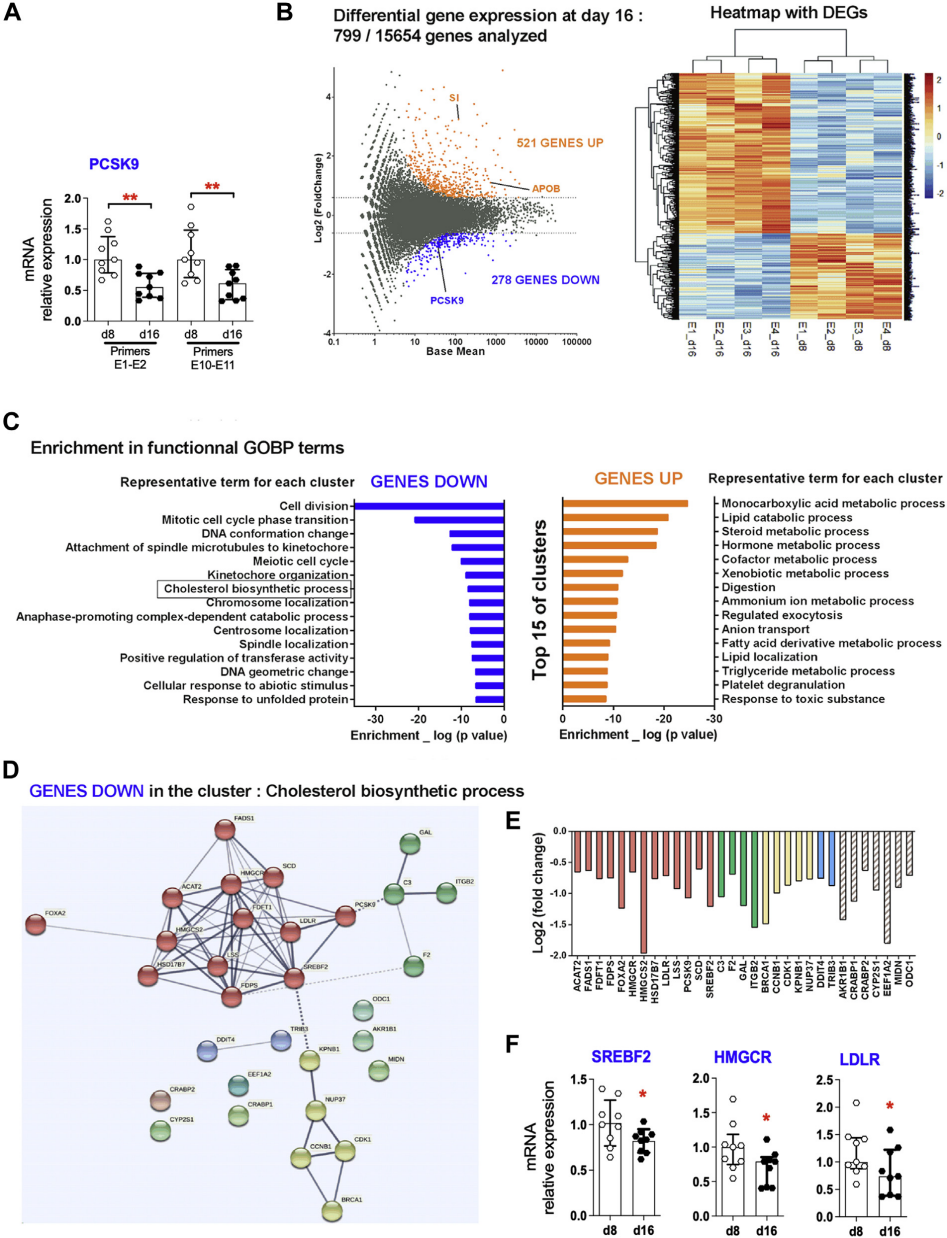
SREBP2-regulated genes, including its own gene *SREBF2* and *PCSK9*, are downregulated in mature Caco-2 cells. Caco2 cells were recovered at d8 and at d16 after a 2-h secretion assay, and mRNAs were extracted. A: PCSK9 expression relative to that of cyclophilin was analyzed by RT-qPCR in samples from 9 independent experiments. Scatter dot plots + bars (median ± interquartile) are presented. ∗∗*P*< 0.01. B: 3′ Digital gene expression RNA sequencing was performed in samples from 4 independent experiments (E1 to E4) at d8 and d16. MA plot is shown in the left panel: dots corresponding to genes that are significantly upregulated at day 16 (GENES UP), downregulated at day 16 (GENES DOWN), or unchanged are colored in orange, blue, and gray, respectively. A heat map with differentially expressed genes (DEGs) is shown in the right panel: each column represents one sample showing the intensity of the expression profile per gene. C: Bar chart of functional GOBP (gene ontology biological process) terms enrichment analysis for GENES UP and for GENES DOWN is shown. D: Protein-protein interactions for the downregulated genes belonging to the cluster “Cholesterol biosynthetic process” are shown. Line thickness indicates the strength of data support. Four clusters with connected nodes are distinguished according to color of bubbles. E: Log2(fold change) for each downregulated gene of the cluster “Cholesterol biosynthetic process” is indicated. F, SREBF2, HMGCR, and LDLR expression relative to that of cyclophilin was analyzed by RT-qPCR in samples from nine independent experiments. Scatter dot plots + bars (median ± interquartile) are presented. ∗*P*< 0.05. HMGCR, HMG-COA reductase; LDLR, LDL receptor; PCSK9, proprotein convertase subtilisin/kexin type 9.

We next compared gene expression levels in Caco2 cells after 8 and 16 days of differentiation. Whereas *SUCROSE ISOMALTASE* and *APOB* expression, two markers of enterocyte maturation, were increased in Caco2 cells at day 16 (Fig. 1C), a parallel significant decrease of PCSK9 mRNA expression was observed by qPCR (−50%) (Fig. 3A). Using a differential 3′DGE RNA analysis, we confirmed these results: 799 genes were differentially expressed between Caco2 cells at day 8 and day 16 out of 15,654 genes analyzed (Fig. 3B). *PCSK9* was found in the list of genes significantly downregulated at day 16 (Fig. 3B), thus confirming the above data. Interestingly, analysis of downregulated genes in Caco2 cells at day 16 revealed an enriched functional cluster related to the cholesterol biosynthetic process, as well as various clusters related to cell division and cell cycle processes (Fig. 3C, supplemental Tables S2 and S3).

Besides, genes upregulated in mature Caco2 cells were related to metabolism of lipids, triglyceride metabolic process, or digestion process, validating the phenotype of nonproliferative mature enterocytes of matured Caco-2 cells acquired at day 16. Finally, among downregulated genes in the functional cluster related to the cholesterol biosynthetic process, we distinguished various genes directly under the control of the factor of transcription SREBP2 including its own gene *SREBF2*, *HMG-COA reductase*, *LDLR*, and *PCSK9* (Fig. 3D, E). Decreases in *SREBF2*, *HMG-COA reductase*, and *LDLR* expression were confirmed by qPCR analysis (Fig. 3A, F). In contrast, the expression levels of putative regulators of *PCSK9* gene expression, such as *HNF1-α*, *PPAR-α*, *HNF4-α*, as well as the PCSK9-binding protein *ANNEXIN* *A2*, were not significantly regulated (supplemental Fig. S2 and data not shown).

Altogether, these observations indicate that the loss of PCSK9 secretion seen during the maturation of enterocytes occurs concomitantly with the downregulation of SREBP2-regulated genes, including *PCSK9*.

### Human and mouse intestinal explants does not secrete PCSK9

**Fig. 4:**
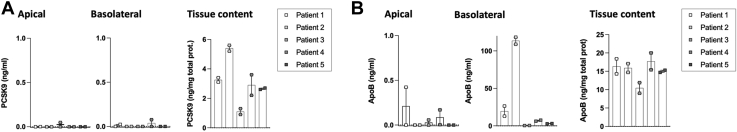
Human intestinal explants do secrete ApoB but not PCSK9. The epithelial layer from human colon biopsies were mounted in Ussing chambers and incubated for 2 h in an oxygenated Krebs solution at 37°C. After incubation, basolateral and apical media and tissues were collected. A: PCSK9 and (B) ApoB protein levels were measured by ELISA. Scatter dot plots + bars represent the median ± interquartile (two biopsies per patient, five patients in total). PCSK9, proprotein convertase subtilisin/kexin type 9.

We next assessed the ex vivo ability of intestinal cells to secrete PCSK9. Nontumoral colon tissue biopsies harvested in patients with colorectal adenocarcinoma were mounted on Ussing chambers and incubated for two hours in an iso-osmotic buffer. After the incubation, basolateral and apical media were collected and intestinal explants were homogenized in nondenaturant conditions. We next quantified by ELISA PCSK9 and ApoB protein concentrations in the different compartments. For all patients (n = 5), a significant amount of PCSK9 protein was measured in the tissue homogenates (Fig. 4A), but we were unable to detect human PCSK9 secretion in both basolateral and apical media (Fig. 4A). As control, we also quantified ApoB expression and secretion. As expected, ApoB protein was well expressed in lysates from intestinal biopsies (Fig. 4B). More importantly, despite the absence of specific stimulation in our experimental conditions, we were able to detect the presence of ApoB in the basolateral media (Fig. 4B), suggesting that the protein secretory pathways were preserved in our biopsies. Ex vivo PCSK9 secretion was also tested using intestinal biopsies (proximal, medial, distal intestine, or colon) from WT or *Ldlr*-deficient mice. Although we readily confirmed PCSK9 protein expression in intestinal explant homogenates, we were unable to detect PCSK9 secretion in both basolateral and apical media from Ussing chambers. To rule out the possibility that the ELISA detection method lacked sensitivity, we also performed acetone precipitation and WB analysis from basolateral media and confirmed the absence of secreted PCSK9 proteins (data not shown).

### The small intestine does not significantly contribute to circulating PCSK9 levels

**Fig. 5:**
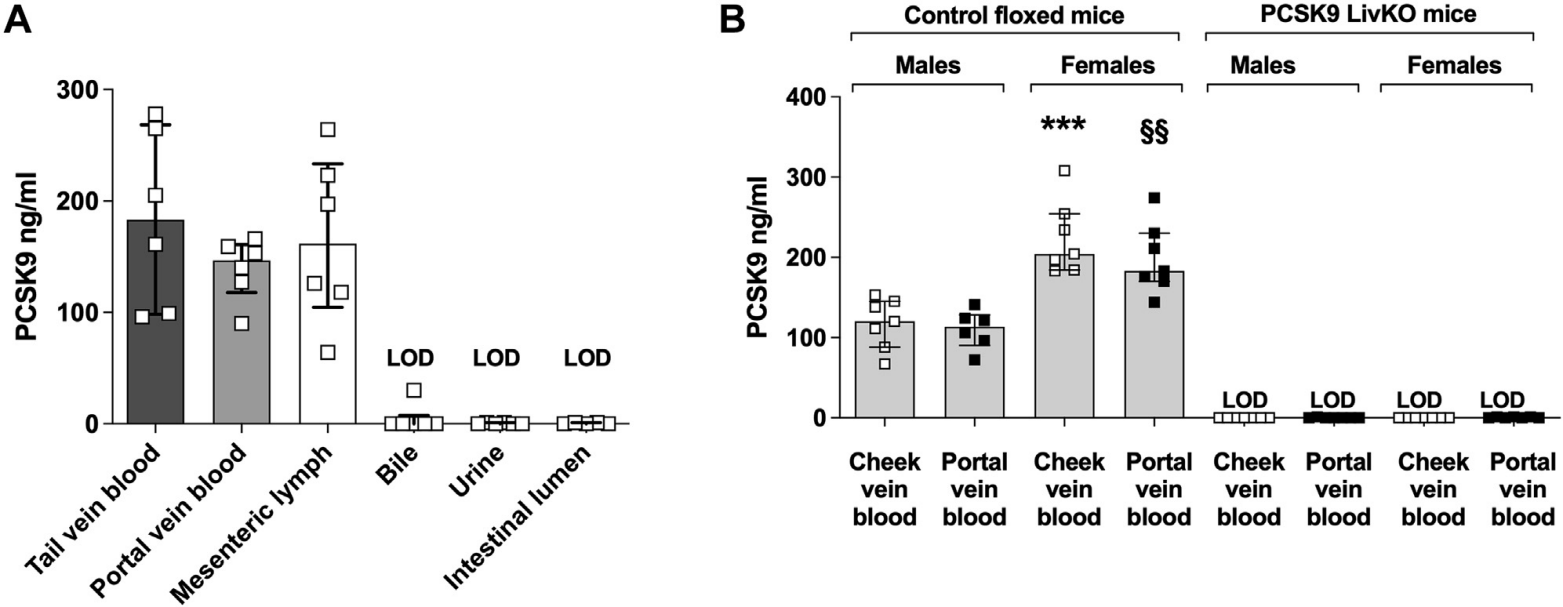
Comparison of circulating PCSK9 concentrations in several biological compartments. A: PCSK9 concentrations in plasma collected from the tail or portal or mesenteric lymph, bile, urine, and intestinal lumen from C57BL6/J female mice were measured by ELISA. B: PCSK9 concentrations in plasma from cheek vein blood and portal vein blood from PCSK9 LivKO mice or control floxed male and female mice. Scatter dot plots + bars represent the median ± interquartile. ∗∗∗*P* < 0.001 versus cheek vein blood in males. §§*P* < 0.01 versus portal vein blood in males. PCSK9 LivKO, mouse model lacking PCSK9 specifically in hepatocytes; PCSK9, proprotein convertase subtilisin/kexin type 9.

Finally, to verify in vivo whether the intestine can partially contribute to circulating PCSK9 levels, we collected fluids from different biological compartments in postprandial conditions (portal blood, mesenteric lymph, intestinal lumen perfusate, bile, urine) and compared their plasma PCSK9 concentrations with those found in peripheral blood (tail vein). First, we did not detect PCSK9 in intestinal lumen perfusate, urine, and bile (Fig. 5A). More importantly, similar PCSK9 concentrations were measured in systemic tail vein blood, portal vein blood, or in mesenteric lymph. In addition, we found the same PCSK9 levels in portal or cheek vein blood from male or female control floxed mice (*Pcsk9f/f*) and PCSK9 LivKO mice (*Pcsk9f/f*; Tg(Alb-cre)+/0) (Fig. 5B). We verified as previously published (15) that circulating PCSK9 is significantly higher in female than male mice (Fig. 5B). Consistent with the Zaid *et al.* study (6), PCSK9 was not detected in systemic or portal vein blood from PCSK9 LivKO male and female mice (Fig. 5B). Altogether, these data confirmed that the expression of PCSK9 in the small intestine does not significantly contribute to circulating PCSK9 levels, suggesting it may act intracellularly or in a paracrine fashion in immature specific intestinal cells.

## Discussion

The aim of this study was to determine whether intestinal cells can secrete PCSK9. This issue was raised by apparently contradictory results from two independent studies regarding the ability of the enterocyte to secrete PCSK9 (6, 11). While in vivo data from PCSK9 LivKO mice indicated that the liver is the sole organ contributing to circulating PCSK9 levels (6), an in vitro study showed that human intestinal Caco2 cells are able to secrete a significant amount of PCSK9 into the basolateral media (11). Furthermore, these studies did not address the hypothesis of a local intestinal secretion of PCSK9 in the portal vein that could directly target the hepatic LDLR and affect cholesterol homeostasis.

Here, we confirm previously published results (6, 11) and extend our knowledge regarding the contribution of the intestine to PCSK9 secretion. *In vivo*, we show that PCSK9 is absent in the systemic and portal blood from PCSK9 LivKO mice. These results confirm those obtained by Zaid *et al.* in the same mouse model by using coimmunoprecipitation and WB to measure PCSK9 in the plasma (6). *In vitro*, we provide a potential mechanistic explanation by showing that human intestinal Caco2 cells progressively lose their ability to secrete PCSK9 upon differentiation. In accordance with these data, the secretion of PCSK9 was found to be significantly reduced in murine primary differentiated epithelial intestinal cells compared with intestinal stem cells. Altogether, our data reconciliate the existing articles and clearly demonstrate that the small intestine does not contribute to plasma PCSK9 levels. In agreement with the present article, we recently showed that plasma PCSK9 levels are similar in control floxed and intestinal *Pcsk9*-deficient mice (10). Finally, even the hypothesis for an autocrine or paracrine secretion of PCSK9 seems unlikely regarding the absence of PCSK9 in the basolateral media of Ussing chambers from human and mouse tissues. However, PCSK9 protein is still present at significant levels intracellularly in intestinal cells, and we cannot completely exclude that under specific stimuli, the small intestine can release PCSK9 into the portal compartment.

Our data suggest that the loss of ability of mature Caco2 cells to secrete PCSK9 is mainly attributable to a significant transcriptional reduction of *PCSK9*. Transcriptomic analysis revealed that *SREBP2* gene expression and SREBP2 target gene expression were robustly decreased in Caco2 cells after 16 days of differentiation. Additional complementary approaches would be required to determine whether the SREBP2 pathway is the sole transcription factor responsible for the decrease of *PCSK9* expression in mature enterocytes. However, results from quantitative PCR showed at least that the expression of *HNF1α*, *HNF4α*, and *PPARα* remain similar between immature or mature Caco2 cells.

We cannot completely rule out the hypothesis that post-transcriptional mechanisms can participate to a lesser extent to the loss of PCSK9 secretion in mature enterocytes. Indeed, apical PCSK9 secretion appears to be less affected by cell differentiation than basolateral PCSK9 secretion. However, the molecular determinants involved in the intracellular trafficking of PCSK9 and its secretion remain to date poorly documented. PCSK9 is secreted by the classical pathway including the reticulum-Golgi axis, and three proteins have been described to influence PCSK9 secretion: Sec24a, a protein belonging to the COPII complex (16), GRP94 is a resident ER protein involved in protein folding (17), and annexin A2, a calcium-regulated membrane-binding protein (18). Our data showed that *SEC24A* gene expression was indeed downregulated at day 16 compared with day 8 (data not shown from differential 3′DGE RNA analysis), but EndoH experiments show that PCSK9 ER-to-Golgi trafficking remains functional regardless of the state of Caco2 differentiation. Both GRP94 and annexin A2 expression remains stable along differentiation (supplemental Fig. S2 and data not shown), arguing against a potential involvement in PCSK9 secretion.

The physiological and functional relevance of the biphasic secretion of PCSK9 observed along Caco-2 cells maturation remained to be demonstrated in vivo. Despite technical challenges, it would be interesting to determine in vivo whether the immature intestinal cells found in the crypts can efficiently secrete PCSK9 and the consequence of PCSK9 deletion or inhibition specifically in the crypts. The interest of such an approach is supported by our data showing that the secretion of PCSK9 by murine primary intestinal cells is affected by their maturity status. Although intestinal *Pcsk9*-deficient mice did not present altered plasma PCSK9 levels (10), *villin*-CRE mice that we used to target PCSK9 deletion in intestinal epithelial cells did not allow to induce full gene deletion in cells from the crypt. Thus, further studies will be required to emphasize the importance of PCSK9 secretion in immature intestinal cells.

The disconnection in the gut between the significant intracellular presence of PCSK9 in the gut and its undetectable secretion is an important observation that opens up interesting perspectives to the understanding of PCSK9 biology. We have previously demonstrated that several intestinal functions such as the TICE and PPL are affected by PCSK9 (8, 9). The present data suggest that these pathways are not affected by autocrine/paracrine intestinal secretion and are in agreement with the finding that intestinal PCSK9 deficiency does not alter lipid homeostasis and notably PPL in mice (10) and that liver-derived circulating PCSK9 rather than intestinal PCSK9 is a critical regulator of intestinal lipoprotein metabolism regulation (10).

In conclusion, we demonstrated that PCSK9 is not secreted from mature enterocytes and does not contribute to circulating PCSK9 found in systemic and portal blood. Thereby, the role of intracellular PCSK9 in enterocyte still remains a mystery and additional studies are warranted to solve this intriguing question.

## Data availability

High-throughput data from 3′DGE RNA sequencing analysis are available in GEO database with the following accession number: GSE162633.

## Supplemental data

This article contains supplemental data (14).

## Figures and Tables

**Figure:**
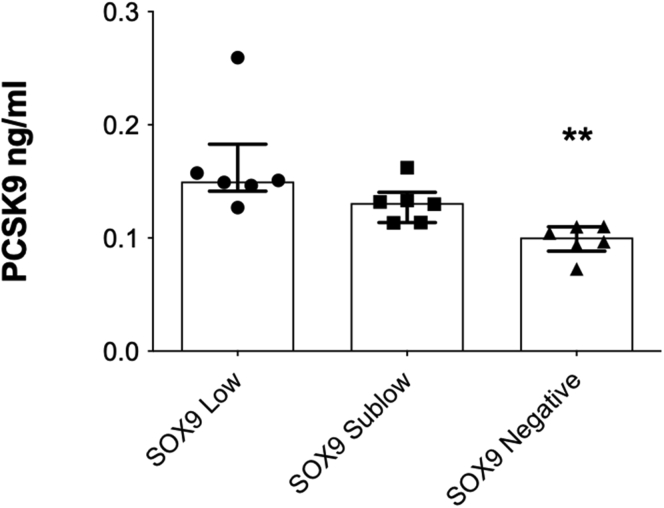
Supplemental Fig. S1PCSK9 secretion is reduced in differentiated murine primary intestinal cells compared with intestinal stem cells.PCSK9 secretion was measured inthree Sox9-EGFP cell populations (Sox9-EGFP negative, Sox9-EGFP sublow, and Sox9-EGFP low cells) after 24 h of culture. Scatter dot plots + bars represent the median ± interquartile of 6 independent experiments.∗*P*< 0.05.PCSK9, proprotein convertase subtilisin/kexin type 9.

**Figure:**
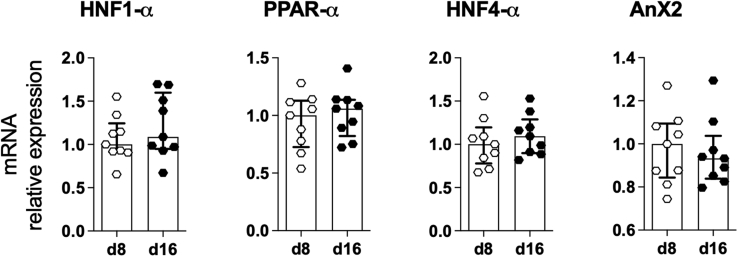
Supplemental Fig. S2HNF1-α, PPAR-α, HNF4-α,and Anx2 are not differentially expressed between immature and mature Caco-2 cells.Caco-2 cells were recovered at day 8 (d8) and day 16 (d16) after 2 h of secretion, and mRNA from cells were extracted. Analysis of HNF1-α, PPAR-*a*, HNF4-*a*,and Anx2gene expression was done by RT-qPCR with samples fromnineindependent experiments. Scatter dot plots+bars represent the median±interquartile of expression levels for each gene relative to cyclophilin gene expression levels.
