# Supplementary material for: PCSK9 is not secreted from mature differentiated intestinal cells
Source: J Lipid Res. 2021 Jul 17;62:100096. doi: 10.1016/j.jlr.2021.100096 (PMC8436166; doi:10.1016/j.jlr.2021.100096)
Supplement: Supplemental Materials and Methods [file mmc1.docx]

Supplemental MATERIALS and METHODS :

**Isolation, culture of Sox9-EGFP cell populations.**

Isolation and culture of the three Sox9-EGFP cell populations (Sox9-EGFP Negative, Sox9-EGFP Sublow and Sox9-EGFP Low cells) were carried out following methods described previously (14). Briefly, jejunum of 6–10-week-old Sox9-EGFP mice were flushed with ice cold 1X PBS, cut open longitudinally, and placed in 30mM EDTA/1.5mM DTT/ PBS over ice for 15 minutes, and then incubated in 30mM EDTA/PBS at 37C for 8 minutes. Jejunal tissue was shaken vigorously and intact tissue was discarded. Remaining cells were washed with 1X PBS and incubated in 0.3U/ml dispase (Corning, NY)/ Hanks buffered saline solution (HBSS) at 37C. Samples were shaken vigorously every 2 minutes for 10 minutes, and fetal bovine serum (FBS, 10% v/v) (Gemini, West Sacramento, CA) and 100g/ml DNase I (Roche, Basel, Switzerland) were subsequently added. Prior to FACS, dissociated cells were sequentially passed through 100m, 70m, and 40m filters. The cells were pelleted and placed in Advanced DMEM F12 (Life Technologies, Carlsbad, CA)/ 10% FBS/ 100g DNaseI/10M Y27632 (Sigma, St. Louis, MO). Sorting of Sox9-EGFP Low, Sublow and Negative cells was performed using a Sony SH800 cell sorter and software (Sony Biotechnology Inc., San Jose, CA). Dead and immune cells were excluded using forward-side scatter gating, and doublets were discriminated using forward scatter and back scatter height-width plots. Gating strategy was identical to previous studies (14). Sorted Sox9-EGFP cells (Sox9-EGFP Negative, Sox9-EGFP Sublow, and Sox9-EGFP Low cells) were resuspended at a density of 20,000 cells/50l/well (24-well plate) in hESC-qualified Matrigel (Corning) supplemented with 1M Jagged-1 peptide (AnaSpec, San Jose, CA), 50ng/ml mEGF (R&D, Minneapolis, MN), 100ng/ml Noggin (Peprotech, Rocky Hill, NJ), and 1g/ml R-Spondin 1 (R&D). After total polymerization, droplets were overlaid with 500l Advanced DMEM/F12 containing N2 supplement (Life Technologies), B27 supplement minus vitamin A (Life Technologies), 10mM HEPES (Life Technologies), 50IU/ml penicillin (Gemini), 50g/ml streptomycin (Gemini), 2mM L-glutamine (Corning), 1M Jagged-1 peptide, 50ng/ml mEGF, 100ng/ml Noggin, 1g/ml R-Spondin 1 and 10M Y27632. Twenty-four hours post-plating, supernatants were collected, centrifuged for 5 minutes at 3,500rpm to eliminate potential cells or debris, and snapfrozen in liquid nitrogen. PCSK9 levels were determined in supernatants using ELISA kit CY-8078 from Circulex according to manufacturer’s instructions.
