# Supplementary material for: PCSK9 is not secreted from mature differentiated intestinal cells
Source: J Lipid Res. 2021 Jul 17;62:100096. doi: 10.1016/j.jlr.2021.100096 (PMC8436166; doi:10.1016/j.jlr.2021.100096)
Supplement: Supplemental Table S1 [file mmc2.docx]

| **Gene name** | **Forward (5'-3')** | **Reverse (5'-3')** |
| --- | --- | --- |
| PCSK9 (Exon1 - Exon2) | ACTACGAGGAGCTGGTGCTA | AGAGGTGGGTCTCCTCCTTC |
| PCSK9 (Exon10 - Exon11) | TTTGGGGGTGAGGGTGTCTA | AAGGTCCTCCACCTCCCAG |
| Sucrase Isomaltase | CGTTTAACGGACTCCCTCAATTT | TGGCACGTCGACCTATGCA |
| ApoB | GCCAGTTTCCAGGGACTCAA | GTACCGTCCCTACCTCCCTT |
| SREBF2 | CTGGTATATCAAAGGCTGCTGGAT | ACGGTAATGATCACGCCAACA |
| HMGCR | AGGCTGCAGAGCAATAGGTCGTCTT | TTCGAGCCAGGCTTTCACTTC |
| LDLR | AAGGCTGTCCCCCCAAGAA | CGAACTGCCGAGAGATGCA |
| HNF1-α | GTCCACGGTGTGCGCTATG | GCTTGGCTTCTGTACTCAGC |
| PPAR-α | GAAGCTGAACCACCCTGAGT | ACGTGTTCCGTGACAATCTGT |
| HNF4-α | GGGATCAACGGCGACATTC | AGCTGCTCCTTCATGGACTCA |
| AnX2 | GAGCGGGATGCTTTGAACATT | TAGGCGAAGGCAATATCCTGF |
| Cyclophilin | GCATACGGGTCCTGGCATCTTGTCC | ATGGTGATCTTCTTGCTGGTCTTGC |

**Supplemental Table 1. Sequences of primers used in qPCR analyses**
