# Supplementary material for: PCSK9 is not secreted from mature differentiated intestinal cells
Source: J Lipid Res. 2021 Jul 17;62:100096. doi: 10.1016/j.jlr.2021.100096 (PMC8436166; doi:10.1016/j.jlr.2021.100096)
Supplement: Supplemental Table S2 [file mmc3.docx]

**Supplemental Table 2. GOBP Terms & genes per terms in the cluster "Cholesterol biosynthetic process" for GENES DOWN.**

| **Term** | **Description** | **LogP** | **Genes** |
| --- | --- | --- | --- |
| GO:0006695 | cholesterol biosynthetic process | -8,37 | ACAT2,FDFT1,FDPS,HMGCR,HMGCS2,KPNB1,LSS,SCD,SREBF2,HSD17B7 |
| GO:1902653 | secondary alcohol biosynthetic process | -8,37 | ACAT2,FDFT1,FDPS,HMGCR,HMGCS2,KPNB1,LSS,SCD,SREBF2,HSD17B7 |
| GO:0016126 | sterol biosynthetic process | -8,04 | ACAT2,FDFT1,FDPS,HMGCR,HMGCS2,KPNB1,LSS,SCD,SREBF2,HSD17B7 |
| GO:0008203 | cholesterol metabolic process | -7,30 | ACAT2,FDFT1,FDPS,HMGCR,HMGCS2,KPNB1,LDLR,LSS,SCD,SREBF2,HSD17B7,PCSK9 |
| GO:1902652 | secondary alcohol metabolic process | -7,21 | ACAT2,FDFT1,FDPS,HMGCR,HMGCS2,KPNB1,LDLR,LSS,SCD,SREBF2,HSD17B7,PCSK9 |
| GO:0016125 | sterol metabolic process | -6,83 | ACAT2,FDFT1,FDPS,HMGCR,HMGCS2,KPNB1,LDLR,LSS,SCD,SREBF2,HSD17B7,PCSK9 |
| GO:0090181 | regulation of cholesterol metabolic process | -6,53 | FDFT1,FDPS,HMGCR,KPNB1,LDLR,LSS,SCD,SREBF2 |
| GO:0045540 | regulation of cholesterol biosynthetic process | -6,15 | FDFT1,FDPS,HMGCR,KPNB1,LSS,SCD,SREBF2 |
| GO:0106118 | regulation of sterol biosynthetic process | -6,15 | FDFT1,FDPS,HMGCR,KPNB1,LSS,SCD,SREBF2 |
| GO:0046165 | alcohol biosynthetic process | -6,09 | ACAT2,AKR1B1,FDFT1,FDPS,HMGCR,HMGCS2,KPNB1,LSS,SCD,SREBF2,HSD17B7 |
| GO:0006694 | steroid biosynthetic process | -5,29 | ACAT2,AKR1B1,FDFT1,FDPS,HMGCR,HMGCS2,KPNB1,LSS,SCD,SREBF2,HSD17B7 |
| GO:0008202 | steroid metabolic process | -5,17 | ACAT2,AKR1B1,FDFT1,FDPS,HMGCR,HMGCS2,KPNB1,LDLR,LSS,SCD,SREBF2,GAL,HSD17B7,PCSK9 |
| GO:0019218 | regulation of steroid metabolic process | -5,17 | FDFT1,FDPS,HMGCR,KPNB1,LDLR,LSS,SCD,SREBF2,GAL |
| GO:0062012 | regulation of small molecule metabolic process | -5,13 | BRCA1,CCNB1,CDK1,FDFT1,FDPS,HMGCR,FOXA2,KPNB1,LDLR,LSS,ODC1,SCD,SREBF2,DDIT4,TRIB3,NUP37,MIDN |
| GO:0019216 | regulation of lipid metabolic process | -5,12 | BRCA1,C3,EEF1A2,F2,FDFT1,FDPS,HMGCR,HMGCS2,KPNB1,LDLR,FADS1,LSS,SCD,SREBF2,GAL,TRIB3 |
| GO:0046890 | regulation of lipid biosynthetic process | -5,11 | BRCA1,C3,FDFT1,FDPS,HMGCR,KPNB1,LDLR,LSS,SCD,SREBF2,TRIB3 |
| GO:0045338 | farnesyl diphosphate metabolic process | -5,02 | FDFT1,FDPS,HMGCS2 |
| GO:0006720 | isoprenoid metabolic process | -4,84 | AKR1B1,CRABP1,CRABP2,FDFT1,FDPS,HMGCR,HMGCS2,LSS,CYP2S1 |
| GO:1902930 | regulation of alcohol biosynthetic process | -4,72 | FDFT1,FDPS,HMGCR,KPNB1,LSS,SCD,SREBF2 |
| GO:0006721 | terpenoid metabolic process | -4,46 | AKR1B1,CRABP1,CRABP2,FDFT1,FDPS,HMGCS2,LSS,CYP2S1 |
| GO:0050810 | regulation of steroid biosynthetic process | -4,22 | FDFT1,FDPS,HMGCR,KPNB1,LSS,SCD,SREBF2 |
| GO:1901617 | organic hydroxy compound biosynthetic process | -4,15 | ACAT2,AKR1B1,FDFT1,FDPS,HMGCR,HMGCS2,KPNB1,LSS,SCD,SREBF2,HSD17B7 |
| GO:0006066 | alcohol metabolic process | -3,92 | ACAT2,AKR1B1,FDFT1,FDPS,HMGCR,HMGCS2,KPNB1,LDLR,LSS,SCD,SREBF2,HSD17B7,PCSK9 |
| GO:0008299 | isoprenoid biosynthetic process | -3,79 | FDPS,HMGCR,HMGCS2,LSS |
| GO:0016114 | terpenoid biosynthetic process | -3,39 | FDPS,HMGCS2,LSS |
| GO:1901615 | organic hydroxy compound metabolic process | -2,84 | ACAT2,AKR1B1,FDFT1,FDPS,HMGCR,HMGCS2,ITGB2,KPNB1,LDLR,LSS,SCD,SREBF2,HSD17B7,PCSK9 |
| GO:0008610 | lipid biosynthetic process | -2,54 | ACAT2,AKR1B1,BRCA1,C3,FDFT1,FDPS,HMGCR,HMGCS2,KPNB1,LDLR,FADS1,LSS,SCD,SREBF2,HSD17B7,TRIB3 |
