# Supplementary material for: PCSK9 is not secreted from mature differentiated intestinal cells
Source: J Lipid Res. 2021 Jul 17;62:100096. doi: 10.1016/j.jlr.2021.100096 (PMC8436166; doi:10.1016/j.jlr.2021.100096)
Supplement: Supplemental Table S3 [file mmc4.docx]

**Supplemental Table 3. Genes in clusters of GOBP terms enriched for GENES UP and GENES DOWN sets.**

|  | **Cluster ID** | **Terms** | **Description** | **LogP** | **Genes** |
| --- | --- | --- | --- | --- | --- |
| **GENES DOWN** | Cluster 1 | GO:0051301 | Cell division | -34,73 | BUB1,CCNB1,CDK1,CDC6,CDC20,CDC25C,CENPA,CENPE,CKS1B,CKS2,ECT2,ETV5,KIF11,MAD2L1,NEK2,PLK1,RAD21,RTKN,AURKA,TOP2A,WEE1,HMGA2,FZD7,RUVBL1,PRC1,CCNB2,AURKB,PTTG1,NCAPD2,SMC4,KIF20A,NDC80,KIF2C,UBE2C,TPX2,ITGB3BP,ORC6,UBE2S,RACGAP1,NUSAP1,ANLN,CDCA8,INTS13,THOC2,SPC25,NUP37,BORA,KNSTRN,MISP,SGO2,SGO1,SKA3,ASPM,CENPW,DUSP1,KIF22,KPNB1,MKI67,RANBP1,DLGAP5,MZT1,SUN1,MND1,BNIP3,BRCA1,HJURP,CENPN,XPO1,TAOK1,H10,HMGA1,BRD4,TMEM39A,MYADM,HSPA5,ID1,LDLR,CORO1C,WDR54,PCSK9 |
|  | Cluster 2 | GO:0044772 | Mitotic cell cycle phase transition | -20,75 | BRCA1,BUB1,CCNB1,CDK1,CDC6,CDC20,CDC25C,CDKN3,CENPE,CKS1B,CKS2,DUSP1,HMMR,PRMT1,MAD2L1,MCM7,NEK2,PLK1,PPP2R2A,RAD21,RRM2,AURKA,WEE1,HMGA2,CCNB2,AURKB,DLGAP5,MELK,NDC80,NES,UBE2C,TPX2,CNOT1,BRD4,ORC6,UBE2S,DTL,ANLN,TAOK1,BORA,BTG1,KIF11,MKI67,RANBP1,TOP2A,PTTG1,NUSAP1,INTS13,ECT2,XPO1,PRC1,KIF20A,RACGAP1,KNSTRN,PTPRK,CDK5RAP1,PRR11,RFC4,DDIT4,FBL,RUVBL1,MRGBP,KMT2C,BGN,CLK1 |
|  | Cluster 3 | GO:0071103 | DNA conformation change | -12,61 | CCNB1,CDK1,CENPA,CHD2,H1-0,HMGB3,HMGA1,MCM7,RFC4,BRD2,TOP2A,HMGA2,RUVBL1,NCAPD2,SMC4,ITGB3BP,NUSAP1,GINS2,HJURP,CHD7,CENPN,NAV2,CENPW |
|  | Cluster 4 | GO:0008608 | Attachment of spindle microtubules to kinetochore | -12,07 | CCNB1,CENPE,ECT2,NEK2,AURKB,NDC80,KIF2C,RACGAP1,KNSTRN,SGO1,CENPA,PLK1,RTKN,PRC1,KIF20A,NUSAP1,ANLN,CDC6,AURKA,ORC6,ASPM |
|  | Cluster 5 | GO:0051321 | Meiotic cell cycle | -9,99 | BUB1,CDC20,CDC25C,CKS2,DUSP1,NEK2,PLK1,RAD21,AURKA,TOP2A,PTTG1,NCAPD2,SMC4,SUN1,MND1,SGO2,SGO1,ASPM,KIF22 |
|  | Cluster 6 | GO:0051383 | Kinetochore organization | -8,88 | CENPA,CENPE,DLGAP5,SMC4,NDC80,CENPN,CENPW,RUVBL1,ITGB3BP,HJURP,HMGB3,HMGA1,HMGA2,NPM3,CHD7,INO80C,H1-0,BRD2,MCM7 |
|  | Cluster 7 | GO:0006695 | Cholesterol biosynthetic process | -8,37 | ACAT2,FDFT1,FDPS,HMGCR,HMGCS2,KPNB1,LSS,SCD,SREBF2,HSD17B7,LDLR,PCSK9,AKR1B1,GAL,BRCA1,CCNB1,CDK1,FOXA2,ODC1,DDIT4,TRIB3,NUP37,MIDN,C3,EEF1A2,F2,FADS1,CRABP1,CRABP2,CYP2S1,ITGB2 |
|  | Cluster 8 | GO:0050000 | Chromosome localization | -8,04 | CCNB1,CENPE,KIF22,KPNB1,AURKB,DLGAP5,NDC80,KIF2C,SUN1,CDCA8,CDK1,CENPA,MAD2L1,NEK2,PLK1,AURKA,XPO1,SEC24A,NUSAP1,INTS13,MISP,ASPM,KIF11,RACGAP1,KIF20A,DPCD,RELB |
|  | Cluster 9 | GO:0031145 | Anaphase-promoting complex-dependent catabolic process | -7,99 | CCNB1,CDK1,CDC20,MAD2L1,PLK1,AURKA,AURKB,PTTG1,UBE2C,UBE2S,EEF1A2,HMGCR,LDLR,ODC1,XPO1,HERPUD1,TRIB1,SF3B3,DTL,PBK,TRIB3,PCSK9,HSPA5,BRCA1,UBA2,FANCI,BNIP3,RBBP6,GTPBP1,CNOT1 |
|  | Cluster 10 | GO:0051642 | Centrosome localization | -7,83 | MAD2L1,AURKA,DLGAP5,SUN1,INTS13,MISP,ASPM |
|  | Cluster 11 | GO:0051653 | Spindle localization | -7,49 | CENPA,KPNB1,MAD2L1,PLK1,NDC80,NUSAP1,MISP,ASPM,CDK1,AURKB,RAD21,WEE1,KIF2C |
|  | Cluster 12 | GO:0051347 | Positive regulation of transferase activity | -7,43 | CCNB1,CDK1,CDC6,CDC20,CENPE,CKS1B,CKS2,DUSP5,ECT2,EEF1A2,F2,IGFBP6,NEK2,PLK1,RFC4,HMGA2,TNFRSF10B,AURKB,UBE2C,TPX2,UBE2S,PBK,TAOK1,TRIB3,BORA |
|  | Cluster 13 | GO:0032392 | DNA geometric change | -6,59 | CHD2,HMGB3,HMGA1,MCM7,RFC4,TOP2A,RUVBL1,GINS2,CHD7,NAV2,BRCA1,CDK1,CDC6,RBBP6,RRM2,ORC6,DTL,BOD1L1 |
|  | Cluster 14 | GO:0071214 | Cellular response to abiotic stimulus | -6,58 | AKR1B1,BNIP3,COL1A1,ECT2,HSPA5,NMT1,PTPRK,RELB,SLC2A1,HMGA2,YBX3,TNFRSF10B,AURKB,NOX1,SLC38A2,PBK,BRCA1,DUSP1,FOS,HMGCR,XRCC4,DTL |
|  | Cluster 15 | GO:0006986 | Response to unfolded protein | -6,48 | ATF3,CTH,GFPT1,HSPA5,HSPA13,MANF,HERPUD1,HYOU1,AGR2,FKBP14,CREB3L2,CLPB,CEBPB,TNFRSF10B,PDIA4,TRIB3,SREBF2 |
|  |  |  |  |  |  |
| **GENES UP** | Cluster 1 | R-HSA-556833 | Metabolism of lipids | -23,82 | ABCA1,ACADM,ACADS,ALB,ALDH3B1,ALOX5AP,ALPI,ASAH1,BAAT,CBR1,CYP2C9,CYP17A1,CYP27A1,AKR1C1,AKR1C2,DECR1,EPHX2,FABP2,GALC,GBA,GPD1,HADHB,HADH,HPGD,HSD3B1,HSD3B2,HSD17B2,PHYH,PLD1,PON2,PON3,CTSA,PCYT2,PLAAT4,SCP2,SLC10A2,AKR1D1,SULT2A1,CUBN,DEGS1,AKR1C3,DGAT1,PEX11A,NR1H4,AGPAT1,PMVK,ACOT7,RGL1,PLA2G15,G0S2,HSD17B14,HAO2,CROT,SPTLC3,BDH2,CHPT1,GBA3,LPIN3,SRD5A3,ELOVL7,ACSF2,DGAT2,ACBD5,SUMF1,ENPP7,AGMO,ACADSB,ADH4,ALDOB,APOA4,BTD,ENTPD5,CES1,FBP1,GSTA1,UGT2B7,UGT2B15,UGT2B17,RGN,DHRS9,GLYAT,NAAA,CRYL1,AIG1,AGXT2,CYP4F12,UGT2A3,ACSM2A,RDH10,ACSM2B,APOB,PDGFA,PRLR,FAXDC2,PIGM,SERINC5,ASPA,ASS1,GATM,OAT,SEPHS2,ADI1 |
|  | Cluster 2 | GO:0016042 | Lipid catabolic process | -19,09 | AADAC,ACADM,ACADS,APOA4,APOB,ASAH1,CES1,CYP27A1,DECR1,FABP2,GALC,GBA,HADHB,HADH,PAFAH2,PHYH,PLD1,PNLIPRP2,PLAAT4,SCP2,SORL1,AKR1D1,SULT1E1,SULT2A1,AKR1C3,SMPDL3A,PLA2G15,NAAA,SMPDL3B,HSD17B14,HAO2,AIG1,CROT,BDH2,GBA3,LPIN3,CYP4F12,SRD5A3,ACBD5,ENPP7,ACADSB,ADH4,ALDH1A1,ALDH3B1,ALDOB,APOBEC1,ARG1,ASPA,BCKDHB,ENTPD5,DAO,HGD,KHK,OAT,PON3,TKFC,DHDH,CRYL1,PCYOX1,NAGK,AGXT2,ENTPD8,APOD,DGAT2,EPHX2,B4GALNT2 |
|  | Cluster 3 | GO:0008202 | Steroid metabolic process | -16,51 | ABCA1,AFP,APOA4,APOB,ASAH1,BAAT,CAT,CES1,CYP2C9,CYP17A1,CYP27A1,AKR1C1,AKR1C2,EPHX2,G6PC,GBA,HSD3B1,HSD3B2,HSD17B2,PRLR,SCP2,AKR1D1,SULT1E1,SULT2A1,UGT2B7,UGT2B15,UGT2B17,CUBN,AKR1C3,NR1H4,DHRS9,PMVK,FAXDC2,HSD17B14,LIMA1,SRD5A3,DGAT2,ALB,SLC10A2,DAO,DDC |
|  | Cluster 4 | GO:0042445 | Hormone metabolic process | -15,66 | ADH4,AFP,ALDH1A1,ALDH9A1,CES1,CTSB,CYP2C9,CYP17A1,CYP27A1,AKR1C1,AKR1C2,HSD3B1,HSD3B2,HSD17B2,PON3,RBP4,AKR1D1,SULT1E1,TTR,UGT2B7,AKR1C3,DGAT1,DHRS9,CPQ,HSD17B14,RETSAT,ACE2,SRD5A3,DGAT2,RDH10,IYD,ABCA1,ALDH3B1,APOA4,APOB,ASAH1,BAAT,CAT,DAO,DDC,EPHX2,GBA,GPD1,MAOB,SCP2,SULT2A1,CUBN,AKR7A2,NR1H4,PMVK,FAXDC2,TKFC,NAAA,HAO2,LIMA1,CYP4F12,SLC25A4,DPP4,FGA,FGB,FGG,HADH,PPP3CA,SLC2A2,SRI,VAMP8,ARL2BP,GPC5,PHYH,RBP2,APOM |
|  | Cluster 5 | R-HSA-211859 | Biological oxidations | -14,87 | NAT2,AADAC,ADH4,ALDH1A1,BPHL,CES1,CYP2C9,CYP27A1,EPHX1,GSTA1,GSTA2,GSTA4,MAOB,SULT1E1,SULT1C2,SULT2A1,UGT2B7,UGT2B15,UGT2B17,AKR7A2,NR1H4,GLYAT,CYP2W1,CYP4F12,UGT2A3,ACY3,ACSM2A,CMBL,ACSM2B,BAAT,AKR1C1,PON3,ARG1,ASS1,F7,GBA,PPP3CA |
|  | Cluster 6 | GO:0051186 | Cofactor metabolic process | -10,79 | ADH4,ALDOB,APOA4,BAAT,BTD,CAT,ENTPD5,AKR1C1,AKR1C2,FBP1,GCHFR,GPD1,GPX3,GSTA1,GSTA2,GSTA4,MAOB,MOCS2,PHYH,PRSS1,TCN2,CUBN,AKR7A2,AKR1C3,DGAT1,NAT8,RGN,SLC23A1,GLYAT,PMVK,ACOT7,CROT,BDH2,PPCS,ELOVL7,ACSF2,AMN,DGAT2,ACSM2A,NADK2,ACSM2B,ENPP1,SULT1E1,SULT1C2,SULT2A1,GNS,GLCE,PCYOX1,ADI1,SQOR,ATP1B1,ATP5MC2,COX7C,G6PC,GUCY2C,NDUFB2,TTR,DNPH1,AGPAT1,SMPDL3A,LHPP,ENTPD8 |
|  | Cluster 7 | GO:0097164 | Ammonium ion metabolic process | -9,48 | ACADM,ALDH9A1,APOA4,ASAH1,DAO,DDC,AKR1C1,AKR1C2,GBA,MAOB,PLAAT4,BBOX1,AKR7A2,AKR1C3,SMPDL3A,PLA2G15,SMPDL3B,CROT,SPTLC3,CHPT1,LPIN3,ENPP7 |
|  | Cluster 8 | R-HSA-196854 | Metabolism of vitamins and cofactors | -8,78 | APOB,BTD,AKR1C1,GPC5,GCHFR,MOCS2,ENPP1,PRSS1,RBP2,RBP4,TCN2,TTR,CUBN,AKR1C3,SLC23A1,RETSAT,APOM,PPCS,AMN,NADK2,CBR1,CYP27A1,RGN,CYP4F12,RDH10,SLC2A2,SLC2A8,SLC48A1,AQP11,BDH2 |
|  | Cluster 9 | R-HSA-425407 | SLC-mediated transmembrane transport | -8,77 | ALB,SLC25A4,APOD,SLC26A3,SLC2A2,SLC3A1,SLC5A1,SLC12A2,SLC15A1,SRI,SLC7A7,SLC35A1,SLC7A9,SLCO2B1,ARL2BP,SLC17A5,SLC2A8,SLC40A1,SLC5A11,SLC36A2,SLC5A12,SLC5A9,SLC39A5,ABCA1,APOB,ATP1B1,CAMK2D,CLCN7,MTTP,FXYD3,PSMB10,PSME1,CUBN,ATP8A2,AMN,AQP10,AQP11,ARG1,GABRA2,SLC23A1,PCYOX1 |
|  | Cluster 10 | GO:0006641 | Triglyceride metabolic process | -8,58 | AADAC,APOA4,APOB,APOBEC1,APOH,CAT,FABP2,G6PC,PNLIPRP2,SORL1,DGAT1,RGN,LPIN3,DGAT2,AGMO,ALPI,GPD1,HADHB,PDGFA,PLD1,PCYT2,PLAAT4,NR1H4,AGPAT1,PLA2G15,CHPT1,PIGM,SERINC5 |
|  | Cluster 11 | GO:0007586 | Digestion | -8,51 | ALPI,APOA4,AKR1C1,AKR1C2,FABP2,GUCY2C,PNLIPRP2,PRSS1,RBP4,SI,SLC2A2,SLC2A5,SLC5A1,AKR1D1,LIMA1,MUC13,PBLD |
|  | Cluster 12 | GO:0045055 | Regulated exocytosis | -8,32 | ALB,ALDH3B1,APLP2,APOH,ARG1,ASAH1,CAT,CTSB,CTSD,CTSH,FGA,FGB,FGG,GAA,GNS,GRN,HLA-F,KNG1,LAMP2,LGALS3BP,OLR1,ORM1,PDGFA,PLD1,SERPINF2,CTSA,RAP2B,SELENOP,SLC2A5,SLPI,TTR,TUBA4A,DEGS1,VAMP8,DGAT1,CREG1,RAB3D,ENDOD1,QPCT,DPP7,SVIP,RAB37,TMEM179B,DNASE1,IL18,UBD,CDH17 |
|  | Cluster 13 | GO:1901568 | Fatty acid derivative metabolic process | -7,89 | ALOX5AP,CBR1,CYP2C9,AKR1C1,AKR1C2,EPHX2,GSTA1,HPGD,PON2,PON3,AKR1C3,DGAT1,ACOT7,BDH2,CYP4F12,ELOVL7,DGAT2,ACSM2A,SCP2,DEGS1,AIG1 |
|  | Cluster 14 | GO:0006081 | Cellular aldehyde metabolic process | -7,44 | ADH4,ALDH1A1,ALDH3B1,ALDH9A1,ALDOB,AKR1C1,KHK,AKR7A2,AKR1C3,TKFC,AGXT2,RDH10,FBP1,ENTPD5,G6PC,GAA,GPD1,DHDH,CRYL1,GBA3,NAGK,GNPTG |
|  | Cluster 15 | GO:0009636 | Response to toxic substance | -7,35 | ADH4,ALB,ALOX5AP,APOA4,APOBEC1,ARG1,ASS1,CCND1,BPHL,CAT,CES1,DDC,EPHX1,EPHX2,G6PC,GPX3,GSTA1,GUCY2C,HPGD,MAOB,MSX1,MT1F,PON2,PON3,PPP3CA,RBP4,RGN,SLC23A1,GLYAT,APOM,AQP10,IYD |
